# Retention of Urine and Its Treatment

**Published:** 1909-05

**Authors:** 


### Retention of Urine and its Treatment

[*The Hospital*, February 27, 1909.]

THE danger of completely emptying the bladder in certain cases of retention of urine is probably not sufficiently appreciated by the majority of practitioners in this country, nor is it enough emphasised in the clinical teaching in our medical schools. Yet the fact is referred to by Sir Henry Thompson in his book published in 1888, and modern textbooks on the subject, both English and Continental, warn against such a danger. In cases of acute retention the bladder may be safely emptied without any fear of evil consequences, but it must be clearly understood what is meant by acute cases. Acute retention of urine not only means inability for the first time to pass water, but also connotes no history of increased frequency, of dribbling, pain above the pubes before passing water, or of ammoniacal urine; any of these symptoms will give rise to the suspicion that a chronic retention of urine has already existed unknown to the patient. Nor need there be any fear of emptying the bladder in those cases of long-standing retention where the bladder has never been allowed to remain partially distended for any length of time; that is to say, where the patient has been relieved daily of his residual urine by the catheter from the very beginning. It is with cases of long-standing retention which have never been relieved by the catheter that the danger lies of death within any time from twenty-four hours to three weeks after the removal of the urine. The immediate cause of death is suppression of urine.

The whole urinary system in such patients, from the calyces of the kidney down to the bladder, is one large cavity; the opening of the ureters into the bladder is no longer oblique, and the normal valvular mechanism of this arrangement is gone; the ureters, the pelvis, and the calyces of the kidney are all dilated. As a result of these changes the kidney is turning out urine against pressure, and not under normal conditions. When the urine is suddenly drawn off this pressure is at once lowered, and the lining membrane of the whole tract lacks the support that it has accommodated itself to. As a result the parts become engorged with blood, leading to the injected kidneys and bladder hemorrhages mentioned above.

How then should a case such as this be treated? In the first place the patient should never be attended to in the practitioner's surgery; he should be put to bed either in his own home or in the ward of a hospital. By means of a catheter a small amount of urine should be drawn off, not more than one-third of the whole bladder contents. This amount, of course, cannot be accurately judged. If in an adult the bladder is well above the umbilicus, it probably holds three pints; if but just palpable above the pubes, only some fifteen ounces. An equal amount of warm boric solution may then be run in through the catheter, allowed to mix with the urine, which is probably foul, and then drawn off again. This may be done some three or four times, so that the fluid in the bladder then consists of urine mixed with boric solution. The patient should be kept quietly in bed, and in twelve or even twenty-four hours' time a further quantity may be drawn off, leaving behind less residual urine than had been left before. In this way in the course of some three or four days the bladder may be cautiously emptied with perfect safety. During this time the patient may be on a full diet for a person at rest, the bowels should be well emptied, and some urinary antiseptic may be given. If a catheter cannot be passed and a suprapubic puncture be found necessary the same precautions must be taken, and a small amount only of urine drawn off on the first occasion.

In acute retention there is no danger in rapidly emptying the bladder, and it is, indeed, necessary to relieve the patient of his pain. The treatment of this condition comes under two heads—to assist the patient to pass his water, or to draw it off by means of some instrument. The decision will depend on the cause of the retention and the amount of pain.

The common causes of retention of urine are three: gonorrhea, stricture, and enlarged prostate. In the first of these conditions it is sometimes said that a catheter should not be passed for fear of infecting the posterior urethra; but this objection is not of much weight, for acute retention does not occur in the acute stage of the disorder, and is due usually to a prostatitis supervening on a chronic or subsiding posterior urethritis, and is brought on by some neglect of treatment when the patient thinks he is getting much better. But the catheter in this condition should be used with extreme care. If a soft india-rubber one fail to pass, a No. 12 French coudé catheter can be tried. No doubt a hot bath and morphia will often relieve the patient in this condition; but it is not a treatment over which much time can be spent, as the patient is in extreme pain from a bladder that has never been distended before, and permanent damage may result.

In retention due to stricture the pain is usually not so great and the retention less absolute. It is useful in such cases to have from the start some definite line along which to work. First, an attempt may be made to pass a catheter, but not too much time should be spent over this. If this fails, the patient should have a hot bath and a dose of morphia, and while he is in the bath another attempt at catheterisation may succeed. Even if this does not, the patient will very likely be successful in passing a small amount of urine. He can then be left for, say, an hour, when a general anesthetic should be given and a third attempt made to reach the bladder with a catheter. If this again fails, the bladder must be punctured above the pubes. The best catheters for cases of stricture are the olive-headed French ones, and if morphia is given a dose of half a grain should be used; small doses are useless.

In the third class of cases, the old man with enlarged prostate, there are two methods that can be used, and two only—the catheter and supra-pubic puncture. Baths and morphia are not likely to be successful, and the use of the drug may be dangerous. A soft rubber catheter will often find its way in along the tortuous urethra, and, failing that, the best kind to be used is the coudé or bi-coudé catheter. Whatever the cause of the retention, the immediate after-treatment is the same; keep the patient in bed, put him on a light diet, and get the bowels well open.

